# Nanorulers for Super‐Resolution Microscopy

**DOI:** 10.1002/cbic.70529

**Published:** 2026-09-27

**Authors:** Gerti Beliu, Alan M. Szalai, Guillermo Acuna, Alexey Chizhik, José Ignacio Gallea, Jörg Enderlein

**Affiliations:** ^1^ Faculty of Chemistry and Pharmacy—Bioimaging, Regensburg Ultrafast Nanoscopy (RUN) University of Regensburg Regensburg Germany; ^2^ Département de Physique – Photonic Nanosystems Université de Fribourg – Faculté des Sciences et de Médecine Fribourg Switzerland; ^3^ Centro de Investigaciones en Bionanociencias (CIBION) Consejo Nacional de Investigaciones Científicas y Técnicas (CONICET) Ciudad Autónoma de Buenos Aires Argentina; ^4^ Third Institute of Physics (Biophysics) Georg August University Göttingen Germany; ^5^ Cluster of Excellence “Multiscale Bioimaging: from Molecular Machines to Networks of Excitable Cells” (MBExC) Universitätsmedizin Göttingen Göttingen Germany

**Keywords:** nanorulers, single‐molecule localization microscopy, super‐resolution microscopy

## Abstract

Super‐resolution fluorescence microscopy has advanced into a regime where molecular‐scale organization can be resolved with nanometer precision. In this context, the accurate interpretation of imaging data increasingly depends on well‐defined calibration standards. Nanorulers have emerged as essential tools to validate resolution, quantify labeling performance, and benchmark analytical methods. Current approaches include DNA‐based, protein‐based, and virus‐based nanorulers, each offering distinct advantages in terms of programmability, structural definition, and biological relevance. This minireview provides an overview of these complementary strategies, with a particular focus on recent developments in protein‐based nanorulers enabled by advances in protein engineering and site‐specific labeling. Together, these approaches contribute to establishing reliable calibration frameworks for quantitative molecular bioimaging.

## Graphical abstract

Nanorulers serve as essential calibration standards to validate optical super‐resolution microscopy down to the Ångström scale. Complementary DNA‐, protein‐, and virus‐based scaffolds provide unique strategies to benchmark spatial resolution, evaluate quantitative labeling efficiency, and mini‐mize linkage errors. Together, these platforms establish accurate, reproducible frameworks that bridge molecular bioimaging with structural biology.

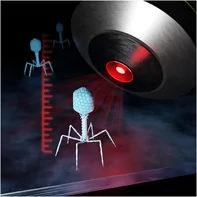

## 1 Introduction

Starting with the invention of Stimulated Emission‐Depletion (STED) microscopy [1, 2], which demonstrated a viable way to circumvent the classical diffraction‐based resolution limit, the field of optical microscopy has undergone a dramatic revolution over the past three decades. However, the rapid proliferation of these advanced modalities has heightened the need for reliable, reproducible, and user‐friendly calibration standards—or “nanorulers”—to validate imaging performance and ensure cross‐system consistency.

Developing such benchmarking samples has been challenging since the early days of super‐resolution microscopy. They are needed not only to evaluate the ultimate resolving power of emerging techniques but also to assess their spatial accuracy, identify distortions or artifacts introduced during image reconstruction, and validate quantitative measurements based on fluorescence intensity or molecular counting. In pioneering works, imaging performance was therefore commonly evaluated using relatively simple or readily available test samples, such as single fluorophores or fluorescent beads randomly immobilized on a glass substrate [3, 4, 5], or by measuring the distances between closely spaced, endogenous biological structures [3, 6, 7]. However, detecting subtle biases or reconstruction artifacts remained difficult, as the ground‐truth spatial distribution of fluorescent labels in these heterogeneous or stochastic samples is extremely challenging to determine a priori. These limitations motivated the development of engineered nanorulers with predefined geometries and well‐controlled labeling patterns, providing an experimentally accessible ground truth against which imaging performance could be quantitatively assessed.

The advent of novel super‐resolution modalities that push spatial resolution limits down to the single‐digit nanometer [8, 9, 10] and, in some cases, even the sub‐nanometer [11, 12] regime, demands exceptionally stable, rigid, and stoichiometric calibration nanorulers. This review provides a comprehensive cross‐section of current state‐of‐the‐art approaches to nanoruler fabrication, focusing specifically on DNA origami architectures, protein‐based scaffolds, and self‐assembling viral particles. We evaluate these distinct platforms based on their structural rigidity, labeling efficiency, and ease of preparation, while highlighting how they address the rigorous demands of localization‐ and intensity‐based nanoscopy.

## 2 DNA‐Based Nanorulers

In the pioneering work that introduced Stochastic Optical Reconstruction Microscopy (STORM), Rust et al. used double‐stranded DNA (dsDNA) as a nanoruler by binding Cy3–Cy5 pairs to specific bases and immobilizing the constructs on a glass surface [13]. Although this represented a significant advance over randomly distributed dyes or biological samples, it did not yet constitute an ideal standard. These constructs are limited to one dimension, and the inherent flexibility of dsDNA prevents precise knowledge of the exact distance between dyes bound to a single helix [14]. This limitation becomes even more pronounced for longer DNA molecules carrying multiple fluorophores. Moreover, the unpredictable three‐dimensional orientation of these samples posed a major bottleneck for 2D super‐resolution techniques, as the projected xy‐distance deviates from the nominal design distance whenever the DNA molecule is not parallel to the imaging plane.

Concurrently, the field of DNA nanotechnology was expanding exponentially, eventually providing a solution to most of these limitations. The field was paving the way toward the large‐scale fabrication of nanoscale objects [15, 16, 17], with DNA origami playing a central role [18, 19, 20]. This technique quickly gained widespread attention across disciplines, as it offered a unique means to construct rigid, stable structures with dimensions ranging from tens to hundreds of nanometers, each capable of incorporating specific modifications at predefined locations. Consequently, DNA origami rapidly found applications in fields such as sensing, imaging, drug delivery, molecular computing, plasmonics, and nanopatterning [21, 22, 23, 24, 25, 26, 27, 28, 29]. While several excellent review articles comprehensively describe this field and its evolution over the last two decades [30, 31, 32, 33], the focus of this review is strictly on the role of DNA origami in super‐resolution microscopy, where it serves as an indispensable calibration standard [34, 35, 36].

**FIGURE 1:**
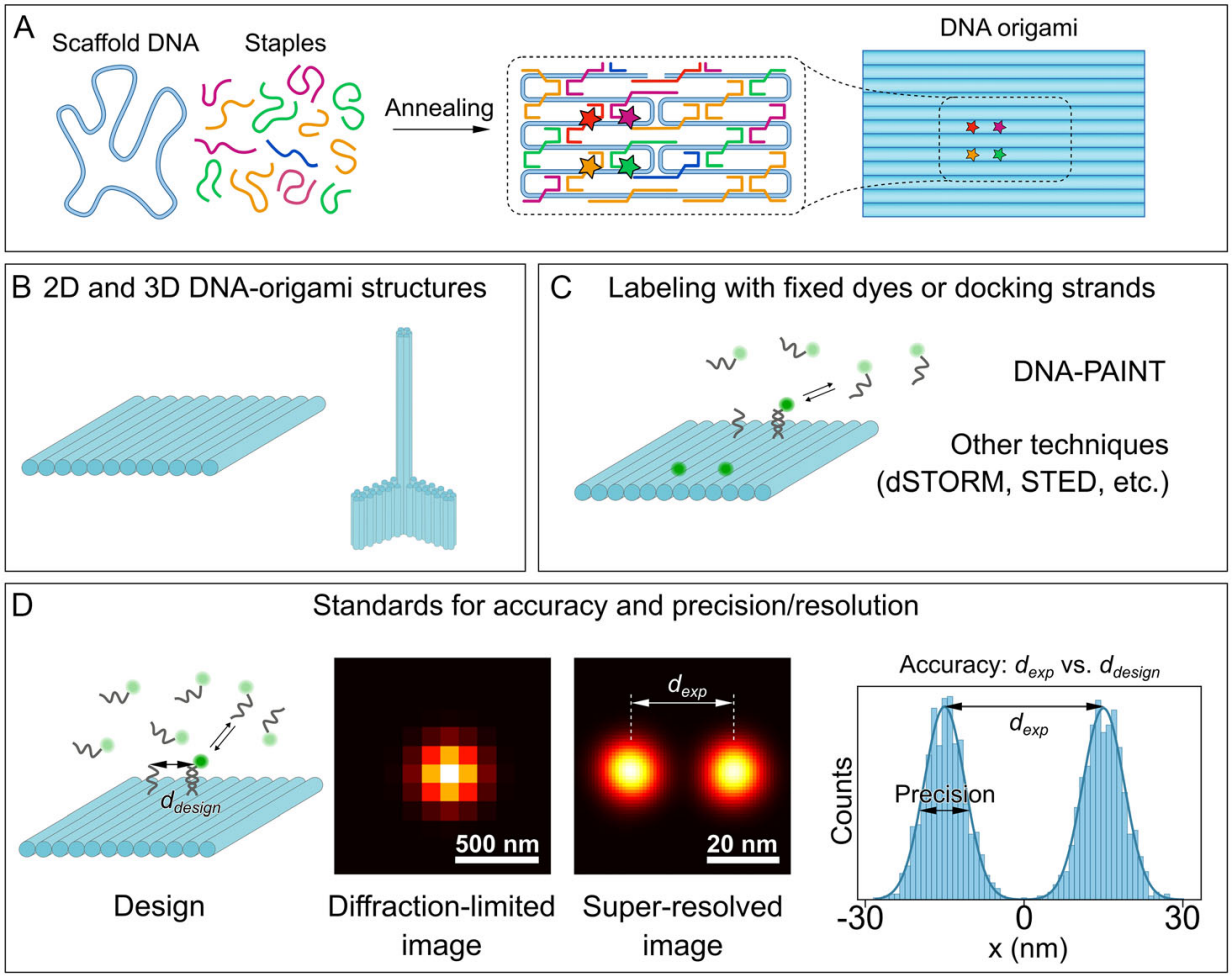
Design principles of DNA origami and its use as nanorulers. (A) Sketch of DNA origami folding process. A closed DNA scaffold is mixed with an excess of short staples. The annealing step takes place under a temperature ramp, after which the DNA origami nanoruler is formed, placing specific modifications on a predefined arrangement. (B) DNA origami nanorulers can be based on flat, two‐dimensional structures, as well as on more complex three‐dimensional structures, the latter being suitable to benchmark 3D super‐resolution methods. (C) DNA origami nanostructures can be modified with docking strands to be used as standards for DNA‐PAINT or fluorescent dyes for other techniques like dSTORM or STED. (D) The well‐known spatial arrangement of docking strands or dyes enables studying both the accuracy and precision/resolution of a super‐resolution technique. The former can be evaluated by comparing the design distances with the measured ones and the latter by measuring the spread of the signal arising from a single dye or docking strand.

DNA origami structures are assembled in a straightforward process: A closed, single‐stranded DNA (the scaffold) is folded by the action of many short single‐stranded DNA molecules (the staples), each containing a fraction complementary to one region of the scaffold and another fraction complementary to a different region (Figure 1A). Using a thermal annealing ramp, this process enables the specific folding of the scaffold into a predefined DNA origami structure with arbitrary 2D or 3D shapes (Figure 1B) within a few hours. It is worth noting that it is a self‐assembled bottom‐up technique that yields billions of structures in parallel, that it can produce structures in two and three dimensions with high degree of flexibility, and that it requires neither highly complex equipment nor demanding chemical steps. For these reasons, it has been widely adopted in many fields of research and nanotechnology.

In particular, the use of DNA origami technology in super‐resolution microscopy began in 2009 [37]; since then, it has become the gold standard for validating newly introduced imaging techniques. Using this platform, researchers can arrange an arbitrary number of fluorophores or docking sites for DNA‐PAINT (DNA points accumulation for imaging in nanoscale topography) at predefined 3D positions across a wide dynamic range, from < 1 nm to hundreds of nanometers (Figure 1C) [38, 39].

Consequently, super‐resolution methods can benchmark their performance against these samples—evaluating spatial resolution by verifying whether the engineered sites are resolvable and assessing accuracy by comparing measured distances to the nominal design values (Figure 1D). Furthermore, because DNA origami structures can be immobilized in a well‐defined orientation on glass surfaces (e.g., via biotin‐avidin chemistry), the measured 2D or 3D distances should closely match the designed values. The remaining error is typically around 0.5–1 nm—often attributable to structural flexibility, dye‐DNA linker length, or nonspecific sticking interactions between the dye and DNA—though it may be slightly higher when limited by the precision and accuracy of the imaging technique itself, which is the most common scenario.

Given the widespread consensus on utilizing DNA origami standards, nearly all super‐resolution methods have demonstrated their capabilities using this approach. From the earliest DNA‐PAINT studies, which resolved target distances of 130 nm, to increasingly powerful implementations—such as DNA‐PAINT variants optimized for higher photon counts to achieve a 5–6 nm spatial resolution [40, 41]—and modern developments like MINFLUX or RASTMIN, where a localization precision of 1–2 nm is attained with only 1000–2000 photons per localization event [8, 42, 43], DNA origami has provided the ideal platform to verify that these methods perform as intended. Remarkably, Ångström‐level resolution and precision have recently been demonstrated with DNA origami samples using RESI (Resolution Enhancement by Sequential Imaging) [11] and MINSTED [9, 44], an achievement that begins to bridge the gap between super‐resolution microscopy and structural biology. Concurrently, three‐dimensional DNA origami structures have proven to be ideal targets for evaluating performance in the perennially challenging axial direction [12, 45, 46, 47, 48]. This high demand for reliable calibration samples has even driven the commercialization of these standards by dedicated suppliers.

In this review, we focus on specific applications where DNA origami has enabled super‐resolution microscopy to advance beyond merely measuring distances on static samples. These examples illustrate how DNA origami has been utilized not only to validate spatial resolution but also to expand the information content of super‐resolution measurements—whether by integrating multimodal approaches, extracting quantitative molecular parameters, validating labeling workflows, or pushing the resolution boundaries toward the limits of structural biology.

**FIGURE 2:**
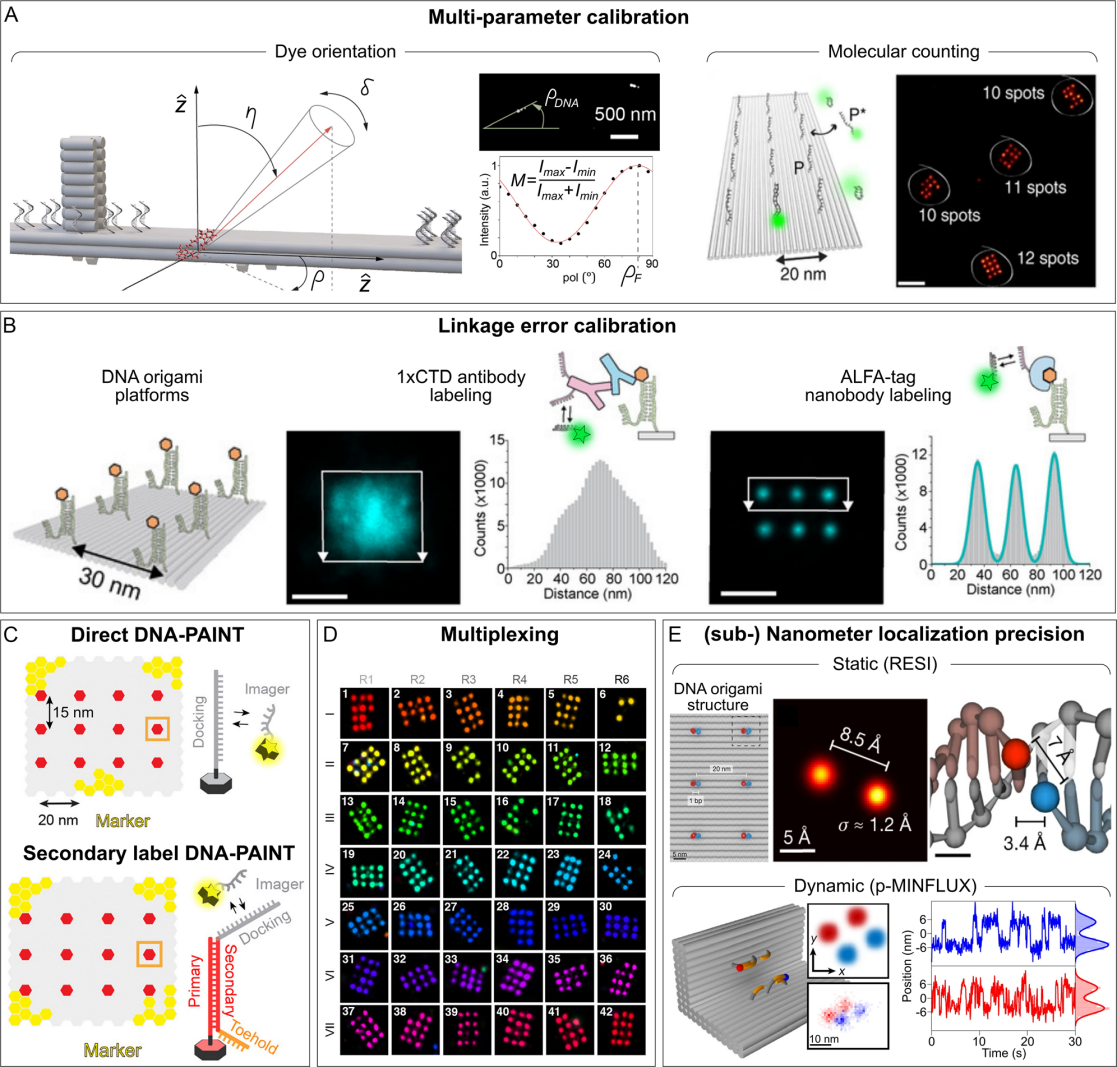
Use of DNA‐based nanorulers in advanced applications. (A) Exemplary cases where the use of DNA origami nanorulers is combined with the measurement of additional parameters. Top: Combined measurement of the orientation of a DNA origami nanostructure and of a fluorescent dye attached to it. Bottom: Example exploiting the number of counted spots with a kinetic analysis of time traces to quantify the number of target sites in DNA‐PAINT experiments. (B) Example where the linkage error of different labeling strategies is directly measured by placing full IgG antibodies and nanobodies on DNA‐based nanorulers at predefined positions. (C) Schematic representation of DNA origami structures used for benchmarking direct versus secondary‐label DNA‐PAINT. (D) Codebook for the 42‐plex screening experiment with an exemplary DNA origami for each barcode, with columns representing barcoding rounds and rows imager‐specific readout rounds. (E) Use of DNA origami structures to benchmark the sub‐nanometer resolution of RESI (top) and to study the single‐digit nanometer resolution of p‐MINFLUX under a dynamic scheme and performing fluorescence lifetime multiplexing (bottom). Elements of this figure were adapted from Ref. [11, 50, 52, 53, 54, 55].

A major frontier beyond simple localization is the simultaneous measurement of additional molecular properties. An illustrative example is the determination of the relative orientation of a single fluorophore with respect to its host nanostructure. Whereas localizing a single emitter yields only its average spatial coordinates, measuring its dipole orientation reveals precisely how it is structurally embedded. By combining polarization‐dependent single‐molecule fluorescence measurements with subsequent DNA‐PAINT imaging on the same DNA origami target, Hübner et al. determined the relative orientation between a fluorophore and the origami scaffold itself [49]. This proof‐of‐principle demonstration paved the way for subsequent studies in which molecular orientation was systematically manipulated by altering the chemical linkage to the DNA origami structure [50, 51] (Figure 2A, left). In these applications, the clear resolution of docking sites is critical because it establishes the spatial reference frame of the nanostructure, transforming the origami from a mere calibration sample into an active vehicle for discovery.

Beyond extracting orientation information, DNA origami has also enabled a quantitative extension of super‐resolution microscopy toward absolute molecular counting. For techniques like DNA‐PAINT, the well‐defined and predictable binding/unbinding kinetics of short DNA sequences make this possible, as the number of localization events scales with the number of docking sites. Using DNA origami structures with precisely known numbers of docking sites to calibrate the expected number of localization events per site (Figure 2A, right), Jungmann et al. developed qPAINT (quantitative DNA‐PAINT) and applied it to quantify the number of Nup98 copies in nuclear pore complexes [52].

A related challenge is determining the practical resolution limits in biologically relevant experiments, which are frequently degraded by factors such as sample drift and linkage error. While drift can be computationally corrected postacquisition, linkage error—the physical displacement between the target protein and its fluorescent reporter—represents a fundamental limitation imposed by the labeling strategy [3, 56]. Ganji et al. utilized a DNA origami platform to directly quantify this effect. By arranging specific antigens in well‐defined geometric patterns and labeling them with either conventional primary/secondary antibody complexes or a single nanobody, they performed DNA‐PAINT to assess the spatial readout (Figure 2B). Their results confirmed that the nanobody introduced negligible linkage error [53]; in contrast, the bulky antibody complexes precluded the resolution of target features spaced closer than 30 nm. This study beautifully demonstrates how a DNA origami platform can isolate and dissect the distinct contributions of individual experimental components—such as labeling topology—from the intrinsic performance of the imaging system.

Regarding sample drift, DNA origami as nanorulers has enabled sub‐nanometer drift correction in DNA‐PAINT experiments [39]. Schnitzbauer et al. proposed a three‐step pipeline where first a redundant cross‐correlation analysis is applied, then individual nanostructures are used as fiducial markers, and finally all individual docking sites from all nanorulers are used as fiducial markers. The success of this strategy heavily relies on counting with a great number of DNA origami structures. For example, the authors show successful experiments using more than 1500 nanostructures. Remarkably, DNA‐based nanorulers can also be exploited to address chromatic aberrations. Using different color channels in single‐molecule localization microscopy (SMLM) may lead to spatially dependent shifts between channels. For this reason, an affine matrix is needed to account for these distortions and perform image corrections. While fluorescent beads can be used for this purpose, a super‐resolved image of a fluorescently labeled DNA origami can provide a higher density of spots, enabling a higher coverage of the field of view [57].

Another major advance in which DNA origami played a crucial validation role is highly multiplexed imaging. Prior to the advent of DNA‐PAINT, multiplexing was typically constrained to a few distinct spectral channels. While sequential imaging utilizing orthogonal DNA strands offers theoretically unlimited multiplexing capabilities [58], practical applications were initially hindered by a limited repertoire of optimized sequences. This bottleneck was recently overcome through the development of secondary‐labeling strategies [55, 59]; in one of these works, Unterauer et al. proposed labeling each target with a unique 20‐nucleotides‐long sequence, called primary barcode (Figure 2C). In a second step, the sample was incubated with a secondary label, containing a sequence complementary to the primary barcode, a speed‐optimized docking sequence, and a 10‐nucleotides long toehold sequence, which was used after imaging for signal extinction through toehold‐mediated strand displacement. Crucially, both the hybridization efficiency and spatial resolution of these new barcodes were first validated using DNA origami platforms (Figure 2D). These benchmarks confirmed that a spatial resolution of 5 nm remained fully attainable, allowing researchers to confidently select high‐efficiency sequences for subsequent multiplexed cellular imaging of up to 30 targets.

The ultimate test of any super‐resolution technique is its capability to resolve the smallest possible distances. Given that typical fluorophores are roughly 1 nm in size, the recent demonstration of Ångström‐level resolution has relied heavily on the sub‐nanometer precise positioning of emitters using DNA origami scaffolds. Among these methods, RESI stands out. Probing its true sub‐nanometer resolving power required a DNA origami structure engineered with six pairs of docking strands separated by only a single base pair, resulting in an in‐plane design distance of 7 Å between adjacent pairs (Figure 2E, left). Remarkably, to resolve such minute features, supplementary docking sites within the origami were utilized as fiducial markers to align localizations from sequential imaging rounds, effectively correcting for lateral translations and rotations. On the other hand, for applications demanding high spatiotemporal resolution—such as tracking proteins in living cells—MINFLUX and its derivatives have emerged as powerful paradigms [60, 61, 62]. To evaluate the tracking capabilities of MINFLUX, DNA origami structures were designed with multiple docking sites adjacent to a single imager strand. The stochastic binding of this imager generated a single‐molecule signal whose centroid shifted over time with sub‐second residence times, effectively acting as a dynamic nanoscale ruler. This versatile platform enabled the experimental validation of 3D p‐MINFLUX utilizing graphene energy transfer (graphene resonance energy transfer, or GRET) [12], the integration of FRET with p‐MINFLUX, and the simultaneous cotracking of two spectrally identical dyes differentiated solely by their fluorescence lifetimes [54] (Figure 2E, right). Across these pioneering studies, the underlying DNA origami structures served as an absolute ground truth for benchmarking molecular‐scale techniques, paving the way for both static and dynamic measurements with resolutions directly approaching those of structural biology.

Finally, it is worth noting that the frontier of super‐resolution microscopy is not limited to pushing the boundaries of maximum spatiotemporal resolution. Paralleling trends in other single‐molecule fluorescence and sensing platforms [63, 64], super‐resolution microscopy has recently been successfully implemented in cost‐effective, portable setups [65, 66]. Smartphone‐based configurations, in particular, hold significant potential for the widespread adoption of super‐resolution techniques in fields such as personalized medicine and point‐of‐care diagnostics by drastically reducing both technical complexity and instrumentation costs. For instance, Loretan et al. demonstrated that a portable setup capable of performing DNA‐PAINT can be constructed for a total budget of under $500. To quantitatively evaluate the achieved spatial resolution of this low‐cost system, they utilized a DNA origami standard to confirm a localization precision of approximately 84 nm. This precision enabled the clear resolution of docking sites separated by only 260 nm—a remarkable benchmarking milestone for such a portable device [65].

In conclusion, DNA origami technology has provided an unparalleled platform for validating major breakthroughs in super‐resolution microscopy. Over the past two decades, it has enabled the precise assessment of both the spatial resolution and accuracy of 2D and 3D imaging techniques. Furthermore, it has been instrumental in benchmarking the integration of super‐resolution methods with complementary biophysical approaches, such as FRET [67, 68, 69], single‐molecule orientation measurements [49, 50, 51], molecular target counting, and highly multiplexed barcoding. Crucially, the recent milestone of sub‐nanometer resolving power was made possible only through the use of DNA origami standards—both for positioning target docking strands at sub‐nanometer proximity and for providing in situ fiducial markers for spatial registration. Given its exceptional programmability, versatility, structural stability, and cost‐effectiveness, DNA origami will undoubtedly remain a key enabler for the development of future super‐resolution modalities and their expanding application across disciplines.

## 3 Protein‐Based Nanorulers

Protein‐based nanorulers occupy a crucial middle ground between fully synthetic DNA nanostructures and endogenous cellular reference targets. As fluorescence microscopy transitions from the 10–20 nm regime toward single‐digit nanometer and even Ångström‐scale precision, ideal calibration standards must combine defined geometry, known fluorophore stoichiometry, negligible linkage error, and high biological compatibility—a set of criteria that no single classical standard satisfies on its own [34, 70, 71].

Classical biological reference structures, such as nuclear pore complexes and microtubules, remain invaluable because they provide native protein architectures with well‐defined dimensions; indeed, they have been instrumental for benchmarking image quality, labeling efficiency, and structural accuracy directly within cells [72, 73]. However, their geometry is inherently fixed rather than programable, and their utility at the molecular scale is ultimately constrained by probe size, steric hindrance regarding labeling accessibility, and significant linkage errors.

**FIGURE 3:**
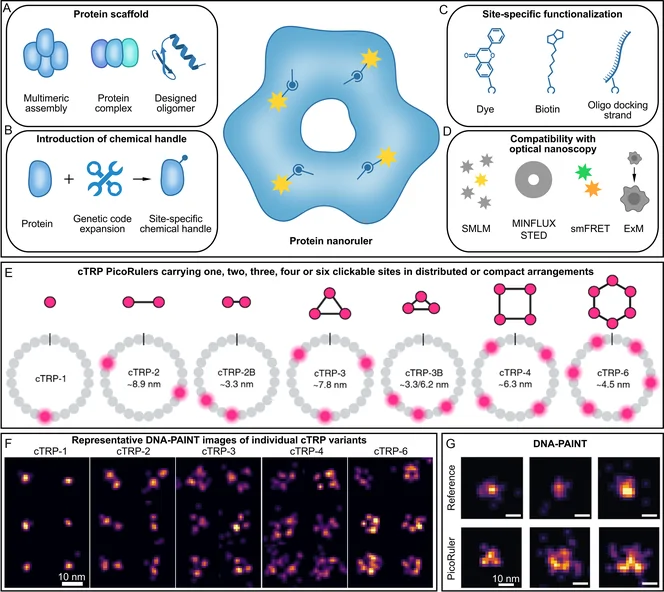
Design principles and key characteristics of protein‐based nanorulers. Protein‐based nanorulers combine the structural definition of engineered protein assemblies with the biological relevance of native macromolecular scaffolds. (A) Suitable protein scaffolds include multimeric protein assemblies, naturally occurring protein complexes, or computationally designed oligomers that provide rigid and well‐defined architectures. (B) Site‐specific chemical handles can be introduced through protein engineering approaches such as GCE, enabling precise fluorophore positioning with minimal linkage error. (C) These handles facilitate the controlled attachment of diverse functional moieties, including fluorescent dyes, affinity tags, and DNA‐PAINT docking strands. (D) As a result, protein nanorulers are compatible with a broad range of optical nanoscopy modalities, including SMLM, MINFLUX, STED microscopy, single‐molecule FRET (smFRET), and ExM. Together, these features establish protein‐based nanorulers as versatile calibration standards that bridge synthetic nanostructures and biologically relevant reference systems. (E) cTRP PicoRulers carrying one, two, three, four, or six clickable sites in distributed or compact arrangements. Approximate designed intersite distances and simplified geometries are shown. (F) Representative DNA‐PAINT images of individual cTRP‐1, cTRP‐2, cTRP‐3, cTRP‐4, and cTRP‐6 particles acquired under identical conditions. (G) DNA‐PAINT images of PCNA. PicoRulers labeled at a low docking‐strand‐to‐PCNA ratio (reference) and a high docking‐strand‐to‐PCNA ratio. Elements of this figure were adapted from Ref. [70, 74].

Conversely, while fully synthetic approaches such as DNA origami provided unmatched control over geometry and fluorophore placement, their in vitro assembly and limited compatibility with complex intracellular environments restrict their utility in natively relevant biological contexts [35, 46, 57]. This is precisely where protein‐based nanorulers emerge as an attractive alternative: They benefit from decades of methodological advances in protein biochemistry, structural biology, and molecular engineering, which collectively enable the de novo design, expression, and site‐specific functionalization of macromolecular scaffolds with exceptional precision (Figure 3A). In contrast to conventional antibody‐based or fusion‐protein labeling, genetic code expansion (GCE) can introduce site‐specific chemical handles with minimal spatial displacement (Figure 3B), which can subsequently be functionalized with fluorophores, affinity tags, or DNA‐PAINT docking strands through bioorthogonal chemistry (Figure 3C) [75, 76]. This modular labeling strategy makes protein nanorulers compatible with diverse fluorescence modalities ranging from SMLM and MINFLUX/STED to FRET and expansion microscopy (ExM) (Figure 3D), and becomes particularly advantageous when the target spatial resolution approaches the physical dimensions of the underlying protein complex itself [77, 78, 79].

In principle, a wide array of peptides and proteins can be considered candidate scaffolds for protein‐based nanorulers (Figure 3A). However, only a distinct subset fulfills the stringent biophysical criteria required at molecular resolution to serve as PicoRulers (protein‐based imaging calibration optical rulers). First, the scaffold must possess a well‐defined, structurally rigid conformation to guarantee precise and reproducible spatial positioning of the attached fluorophores. Second, it must accommodate site‐specific, quantitative labeling at predefined positions, ideally with negligible linkage error. Third, it must exhibit robust biochemical stability and compatibility with diverse experimental conditions, including native intracellular environments.

One of the first experimentally validated protein‐based nanorulers was the PCNA (Proliferating Cell Nuclear Antigen)‐based PicoRuler [70]. The homotrimeric PCNA ring provides a rigid scaffold with intrinsic $C_{3}$ symmetry. GCE was used to introduce one noncanonical amino acid into each protomer, generating three bioorthogonal labeling sites separated by approximately 6 nm. Functionalization with tetrazine‐conjugated fluorophores or DNA‐PAINT docking strands combined defined stoichiometry with minimal linkage error.

Photoswitching fingerprint analysis distinguished PCNA assemblies through differences in fluorophore‐dependent blinking and localization accumulation, whereas DNA‐PAINT provided a spatial readout of the three‐site organization (Figure 3G). PCNA therefore established the feasibility of protein‐based calibration at sub‐10 nm length scales. At the same time, its fixed trimeric symmetry limits the extent to which fluorophore number, spacing, and geometry can be varied independently.

This limitation was recently addressed using circular tandem repeat proteins as programable next‐generation PicoRulers [74]. cTRPs are de novo‐designed protein rings with an outer diameter of approximately 10 nm and repeated surface‐exposed loops that can be modified through GCE and bioorthogonal labeling. This enabled the generation of cTRP PicoRulers carrying one, two, three, four, or six labeling sites in distributed or compact arrangements, with programed intersite distances of approximately 3.3–8.9 nm (Figure 3E).

Systematic variation of fluorophore number and spacing across the cTRP series revealed geometry‐dependent differences in localization accumulation and blinking kinetics, indicating short‐range photophysical interactions. DNA‐PAINT provided an orthogonal single‐particle readout of docking‐site accessibility and programed valency within the approximately 10 nm protein rings (Figure 3F). Together, these measurements establish cTRP PicoRulers as programable standards for assessing labeling stoichiometry, fluorophore accessibility, photophysics, and localization‐based imaging performance [74].

cTRP PicoRulers were also extended beyond purified particles immobilized on coverslips. Recombinant HaloTag tethering and genetically encoded GPI anchoring enabled their presentation at the plasma membrane, providing reference structures in a more biologically relevant environment. In addition, cTRP‐6 PicoRulers with approximately 4.5 nm nearest‐neighbor spacing remained detectable after anchoring, proteolysis, physical expansion, and postexpansion labeling. Protein nanorulers can therefore benchmark not only localization‐based imaging but also labeling accessibility, fluorophore interactions, molecular retention, local expansion, and geometric distortion.

Recent studies in other fluorescence modalities further support the use of engineered proteins as quantitative reference standards, as in the example of a modular protein ladder based on repeated tetratricopeptide motifs for standardizing diverse FRET assays [80]. The constructs generated a predictable range of FRET efficiencies and enabled comparisons across expression systems, labeling strategies, and measurement platforms, including smFRET, flow‐cytometry‐based FRET, and FLIM‐FRET. In ExM, recently a self‐assembling protein nanocage has been utilized to determine the local nanoscale expansion factor [81]. This system revealed differences between macroscopic gel expansion and effective expansion within protein‐rich cellular regions, improving the quantitative interpretation of molecular distances and dimensions in expanded endosomes.

Together, these studies show that protein‐based nanorulers have progressed from fixed natural oligomers to programable and modality‐specific reference systems. In the case of protein‐based nanorulers in combination with GCE and click chemistry, PicoRulers provide a reproducible standard and allow fluorophore number and spacing to be varied within the same compact scaffold. Their defined geometries and compatibility with membrane display may also enable the development of reference standards for molecular tracking, allowing systematic assessment of localization noise, residual drift, fluorophore blinking, missed localizations, and rotational reorientation. Further advances in computational protein design may enable scaffolds with customized dimensions, symmetry, surface chemistry, and cellular localization. Protein nanorulers therefore complement DNA origami, particularly where a protein‐like environment, genetically encoded presentation, or compatibility with cellular and expansion‐based imaging is required.

## 4 Virus‐Based Nanorulers

In the context of nanorulers for superresolution optical microscopy, viral particles emerge as highly attractive biological scaffolds because they are exceptionally stable, reproducible, and biocompatible. Evolved to protect and transport their genomes across diverse environments, viruses combine precise nanoscale architecture with remarkable structural integrity and physicochemical stability [82, 83, 84]. Moreover, they exhibit highly symmetric conformations—typically helical or icosahedral—composed of multiple copies of one or a few distinct coat proteins [85, 86], which can be genetically modified to accommodate precise and versatile fluorescence labeling workflows. While viruses such as the tobacco mosaic virus (TMV) have long served as established standards for distance calibration in electron microscopy [87, 88], comparable, universally applicable biological benchmarks have been notably absent in fluorescence microscopy. Addressing this gap, bacteriophage T4 has recently been introduced as a “3D‐Bio‐NanoRuler” [89], establishing a new paradigm of biological reference standards for super‐resolution light microscopy.

**FIGURE 4:**
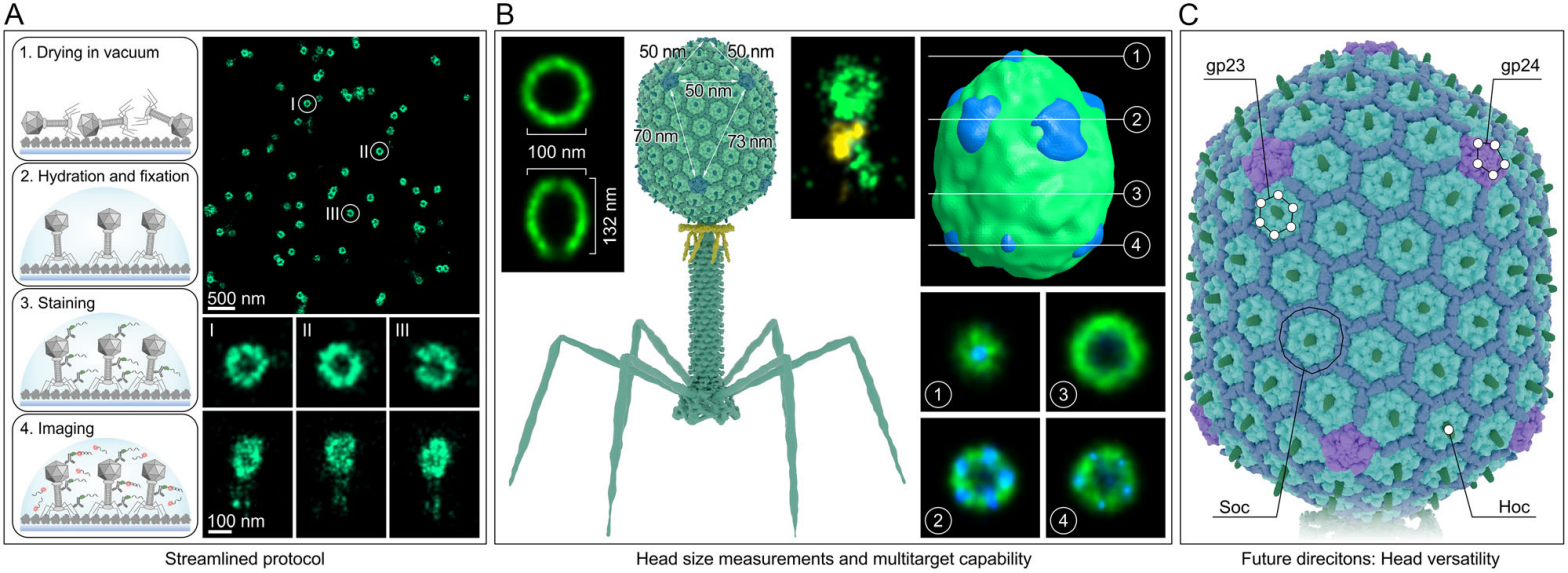
T4 phage as a 3D Bio‐NanoRuler: structural features and labeling sites. (A) Virus sample preparation protocol and representative 3D DNA‐PAINT images of T4 virions. Three individual virions were selected from an x–y cross‐section image. Magnified x–y and x–z projections are shown for each selected virion. (B) Structure‐based cartoon representation of T4 showing the distances between gp24 vertex proteins. Left: Width and length measurements of a T4 capsid obtained by particle averaging (green). Right: Representative two‐color Exchange DNA‐PAINT data of T4 virions targeting distinct structural components. An x–z projection shows the whole T4 virion in green together with the collar protein fibritin in yellow. A 3D rendering of the averaged capsid is shown in green together with gp24 vertex proteins in blue, accompanied by four x–y cross‐sections through the entire structure. (C) Structural model of the T4 capsid highlighting its four structural proteins as potential labeling targets. White dots on gp23 and gp24 indicate the apical protrusions of these proteins. Structural representations were generated using the atomic model of the expanded T4 capsid (PDB ID: 7VS5 [90]) and the cryo‐EM density map of the extended tail (EMDB ID: EMD‐1126 [91]), which were combined and modified for visualization. Elements of this figure were adapted from Ref. [89].

This nonpathogenic virus can be safely managed under Biosafety Level 1 (BSL‐1) conditions, is readily accessible from commercial suppliers, and is straightforward to propagate in high yields. Structurally, it possesses a distinctive ∼260 nm rocket‐shaped architecture characterized by an elongated icosahedral head encapsulating its genomic DNA and a contractile cylindrical tail. The authors developed a streamlined sample‐preparation protocol involving brief vacuum desiccation followed by rehydration and chemical fixation to direct the vertical alignment of the virions onto albumin‐coated glass surfaces (Figure 4A). Consequently, hundreds of individual phages can be imaged concurrently in sharp focus, with the vast majority exhibiting a uniform perpendicular orientation relative to the coverslip. This precise alignment, coupled with the virus’s inherent axial asymmetry—where the head and tail define distinct upper and lower parallel planes—renders it an ideal calibration standard uniquely suited for 3D super‐resolution imaging.

Specifically, the study employed 3D DNA‐PAINT utilizing primary antibodies and secondary nanobodies to label both the entire virion and specific structural capsid proteins. The prolate capsid was characterized through direct spatial measurements and further refined via a particle‐averaging approach, yielding dimensions of 100 nm in width and 132 nm in length (Figure 4B). These experimental results align closely with predicted structural values when accounting for the ∼7 nm linkage error documented for this specific labeling modality [92]. Furthermore, the spatial distribution of the vertex protein gp24 was evaluated by measuring the triangular geometries formed by these labeled corners (Figure 4B); the resulting 3D measurements—56±6nm for the short sides and 72±3nm for the long sides—are in excellent agreement with the established dimensions of the T4 icosahedral facets.

Beyond these specific metrics, the T4 virion features over 40 unique structural proteins distributed across its head, neck, tail, and tail fibers. This extraordinary structural diversity underscores the platform’s multitarget potential, facilitating multichannel calibration alongside robust internal consistency checks. As a proof of concept, two‐color Exchange 3D DNA‐PAINT of the whole virion together with the neck‐associated fibritin protein, gpwac, revealed the expected sub‐capsid localization and collar‐like organization of gpwac (Figure 4B). Notably, the documented capability of T4 to penetrate eukaryotic cells [89, 93] suggests the promising possibility of employing this virus as an in cellulo biological nanoruler for intracellular super‐resolution imaging.

Finally, the study proposes alternative labeling strategies for the four primary capsid proteins to minimize linkage error and facilitate the measurement of significantly shorter distances (Figure 4C). To this end, GCE could be deployed to label the accessory proteins Hoc and Soc (spaced at 14 nm), or to target the apical protrusions of the gp24 homopentamer and the gp23 homohexamer, which are separated by 4.8 nm and 4.5 nm, respectively.

These future developments would enable the rigorous verification of single‐digit nanometer‐scale resolution across all three dimensions. Altogether, the T4 “3D‐Bio‐NanoRuler” represents a highly versatile and much‐needed benchmark for the next generation of 3D super‐resolution microscopy platforms.

## 5 Conclusion and Future Outlook

As fluorescence microscopy advances past the single‐digit nanometer barrier and firmly enters the Ångström‐resolution regime, the absolute reliability of quantitative imaging data shifts from a hardware constraint to a calibration challenge. The accurate extraction of molecular parameters—such as true spatial distances, absolute fluorophore stoichiometry, and single‐molecule dipole orientations—increasingly demands robust, standardized reference frames. The three distinct nanoruler modalities detailed in this review form a powerful, complementary toolkit, yet each exhibits fundamental biophysical trade‐offs that dictate its application landscape.

To systematically guide researchers in selecting the optimal calibration framework for specific nanoscopy implementations, the core attributes, structural benchmarks, and inherent limitations of each platform are summarized in Table 1 below.

**TABLE 1:** Comparison of different nanoruler modalities discussed in this review.

Nanoruler Platform	Structural Basis & Symmetry	**Spatial Range** / **Resolution Benchmarks**	Key Advantages	Primary Bottlenecks
**DNA Origami**	Synthetic, highly programable 2D/3D lattices.	Dynamic range from <1nm to hundreds of nanometers. Enabled 7 Å static testing (RESI).	Unmatched geometric flexibility; commercial availability; ideal for multichannel barcoding and dynamic molecular tracking.	Structural flexibility; sensitivity to high‐salt/divalent cation environments; poor compatibility with native intracellular environments.
**Protein‐Based** (e.g., PCNA or cTRP PicoRulers)	Engineered native or de novo protein scaffolds with fixed or programable geometries.	Sub‐10 nm standards; PCNA provides three ∼6 nm‐spaced sites, whereas cTRPs enable one to six sites with ∼3.3–8.9 nm programed spacing.	Minimal linkage error via GCE and bioorthogonal click chemistry; defined stoichiometry; programable valency and geometry; compatibility with cellular environments.	Multisite incorporation and homogeneous labeling can be technically demanding; labeling accessibility and scaffold orientation require construct‐specific validation.
**Virus‐Based** (e.g., Phage T4)	Rigid, symmetric macromolecular capsids (icosahedral/helical).	Macroscale dimensions (∼260 nm length) with single‐digit Nanometer patterns (4.5–4.8 nm arrays).	Exceptional physical stability; inherent 3D axial asymmetry ideal for vertical *Z*‐axis alignment; high multitarget density (>40 structural proteins).	Large physical footprint can cause steric hindrance; in‐cellulo implementation and structural stability after cellular internalization remain to be established.

A unifying theme across all three platforms is the critical need to decouple the intrinsic resolving power of the optical microscope from the spatial displacement introduced by the labeling strategy—the linkage error. While synthetic DNA origami scaffolds isolate this error using direct fluorophore attachment or short DNA‐PAINT docking linkers, translating these standards into complex biological environments remains notoriously difficult.

Protein‐ and virus‐based nanorulers directly solve this limitation. By shifting away from bulky, traditional antibody complexes (which can introduce  ∼7 nm or more of displacement) toward GCE combined with bio‐orthogonal click chemistry, these biological scaffolds allow for the site‐specific attachment of small organic dyes directly onto the protein backbone. This ensures that the measured optical coordinates accurately reflect the true coordinates of the macromolecular standard, a prerequisite for quantitative structural biology.

Looking forward, the frontier of protein‐based nanorulers lies in further expanding the geometric diversity and biological applicability of programable standards. While PCNA provides a rigid but fixed trimeric scaffold, cTRP PicoRulers demonstrate that de novo‐designed proteins can support variable labeling stoichiometries and defined sub‐10 nm geometries. Further advances in computational protein design may extend this concept toward customized three‐dimensional architectures, surface chemistries, and cellular targeting, combining geometric programmability with the biological compatibility and minimal linkage error of protein‐based standards.

Concurrently, exploiting the ability of virus‐based architectures such as bacteriophage T4 to be internalized by eukaryotic cells opens an exciting avenue for the development of in cellulo biological nanorulers. Such standards will enable researchers to map, quantify, and correct for heterogeneous refractive index variations, molecular crowding, and local intracellular motion within living cells, thereby facilitating the development of appropriate correction strategies.

## Funding

This work was supported by the European Research Council (884488) and Deutsche Forschungsgemeinschaft (EXC 2067/1‐390729940).

## Conflicts of Interest

The authors declare no conflicts of interest.

## Data Availability

Data sharing not applicable to this article as no datasets were generated or analysed during the current study.
